# Role of Argininosuccinate Synthase 1 ‐Dependent L‐Arginine Biosynthesis in the Protective Effect of Endothelial Sirtuin 3 Against Atherosclerosis

**DOI:** 10.1002/advs.202307256

**Published:** 2024-01-17

**Authors:** Xiaoyun Cao, Vivian Wei Yan Wu, Yumeng Han, Huiling Hong, Yalan Wu, Alice Pik Shan Kong, Kathy O Lui, Xiao Yu Tian

**Affiliations:** ^1^ School of Biomedical Sciences Heart and Vascular Institute Faculty of Medicine The Chinese University of Hong Kong Shatin NT Hong Kong SAR 999077 China; ^2^ Department of Chemical Pathology Faculty of Medicine The Chinese University of Hong Kong Shatin NT Hong Kong SAR 999077 China; ^3^ Department of Histology and Embryology School of Basic Medical Sciences Central South University Changsha 410013 China; ^4^ Department of Medicine & Therapeutics Faculty of Medicine The Chinese University of Hong Kong Shatin NT Hong Kong SAR 999077 China

**Keywords:** endothelial cell, sirtuin 3, argininosuccinate synthase 1, L‐arginine synthesis, atherosclerosis

## Abstract

Atherosclerosis is initiated with endothelial cell (EC) dysfunction and vascular inflammation under hyperlipidemia. Sirtuin 3 (SIRT3) is a mitochondrial deacetylase. However, the specific role of endothelial SIRT3 during atherosclerosis remains poorly understood. The present study aims to study the role and mechanism of SIRT3 in EC function during atherosclerosis. Wild‐type Sirt3f/f mice and endothelium‐selective SIRT3 knockout Sirt3f/f; Cdh5Cre/+ (Sirt3^EC‐KO^) mice are injected with adeno‐associated virus (AAV) to overexpress PCSK9 and fed with high‐cholesterol diet (HCD) for 12 weeks to induce atherosclerosis. Sirt3^EC‐KO^ mice exhibit increased atherosclerotic plaque formation, along with elevated macrophage infiltration, vascular inflammation, and reduced circulating L‐arginine levels. In human ECs, SIRT3 inhibition resulted in heightened vascular inflammation, reduced nitric oxide (NO) production, increased reactive oxygen species (ROS), and diminished L‐arginine levels. Silencing of SIRT3 results in hyperacetylation and deactivation of Argininosuccinate Synthase 1 (ASS1), a rate‐limiting enzyme involved in L‐arginine biosynthesis, and this effect is abolished in mutant ASS1. Furthermore, L‐arginine supplementation attenuates enhanced plaque formation and vascular inflammation in Sirt3^EC‐KO^ mice. This study provides compelling evidence supporting the protective role of endothelial SIRT3 in atherosclerosis and also suggests a critical role of SIRT3‐induced deacetylation of ASS1 by ECs for arginine synthesis.

## Graphical abstract

This investigation delves into the role of Sirtuin 3 (SIRT3), a mitochondrial deacetylase, within endothelial cells during atherosclerosis. The findings elucidate that the deletion of endothelial SIRT3 induces hyperacetylation and inactivation of Argininosuccinate Synthase 1 (ASS1), thereby disrupting the crucial L‐arginine synthesis pathway. The reduction in L‐arginine levels significantly contributes to diminished nitric oxide production, exacerbating endothelial dysfunction and atherosclerotic plaque progression.

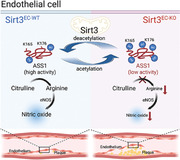

## 1 Introduction

The vascular endothelium is continuously distributed along various types of blood vessels, with its primary function of maintaining normal vascular tone and regulating vascular homeostasis.^[^ ^1^ ^]^ Endothelial dysfunction, which can be triggered by inflammatory mediators, dyslipidemia, or blood flow disturbance is a critical event during the initiation of atherosclerosis.^[^ ^2^ ^]^ Dysfunction of endothelial cells (ECs) impairs the release of vasodilating and protective factors such as nitric oxide (NO), disrupts the balance between vasoconstriction and vasodilation, together with redox imbalance, facilitates EC activation, leading to recruitment and transmigration of immune cells, and platelet aggregation on the vessel wall, eventually leading to vascular inflammation and atherogenesis.^[^ ^3^ ^]^

Many recent research have shown the importance of post‐translational modifications of proteins during the pathogenesis of atherosclerosis.^[^ ^4^ ^]^ Protein acetylation, as one type of these post‐translational changes, plays a critical role in regulating vascular function.^[^ ^5^ ^]^ Sirtuin‐3 (SIRT3), which has higher deacetylase activity compared to SIRT4 and SIRT5, has been identified as the primary protein deacetylase within the mitochondrial matrix, unlike SIRT1 which mainly acts as a nuclear protein deacetylase.^[^ ^6^ ^]^ Inhibition of SIRT3 in vitro or in vivo results in hyperacetylation of mitochondrial, as well as some non‐mitochondrial proteins, affecting protein function and activation, therefore was considered a potential therapeutic target for cardiovascular diseases.^[^ ^6^, ^7^ ^]^

Previous studies using proteomics have identified several mitochondrial enzymes, that are involved in lipid oxidation, inflammation, autophagy, apoptosis, and amino acid metabolism, under the control of SIRT3‐dependent acetylation, mainly in metabolic organs. For instance, the activity of ornithine transcarbamylase (OTC) of the urea cycle, which is involved in fatty acid oxidation was regulated by SIRT3 via deacetylation during dietary restriction, when SIRT3 expression is at a higher level.^[^ ^8^ ^]^ SIRT3 also enhances the ability of the mitochondria to cope with reactive oxygen species (ROS) by directly regulating the acetylation and activity of superoxide dismutase 2 (SOD2) and isocitrate dehydrogenase 2 (IDH2) of the tricarboxylic acid (TCA) cycle, in cardiomyocytes to protect against hypertensive heart failure.^[^ ^9^ ^]^ Additionally, inhibition of SIRT3 exacerbates inflammatory responses through the hyperacetylation of autophagy‐related 5 (ATG5), resulting in impaired autophagosome maturation, and inducing NLRP3 inflammasome activation and EC dysfunction.^[^ ^10^ ^]^ However, most of the studies were done in in vitro cell models or global Sirt3 knockout mice. Considering the different roles of SIRT3 in different cell types, especially those participating in lipid metabolism and immune response, the direct evidence, the specific role, and the downstream protein targets of endothelial SIRT3 in vascular homeostasis and atherosclerosis development, is not entirely clear.

NO, the major vasodilator, also inhibits platelet aggregation, ROS production, and vascular inflammation.^[^ ^11^ ^]^ L‐arginine , which is the substrate for NO production, is a semi‐essential amino acid, derived from diet, intracellular de novo synthesis, and net degradation of intracellular proteins.^[^ ^12^ ^]^ Previous studies showed that the liver of *Sirt3^−/−^* mice has reduced levels of several metabolites involved in the arginine biosynthesis pathway, including citrulline, aspartate, and argininosuccinate.^[^ ^13^ ^]^ Argininosuccinate synthase 1 (ASS1) is the enzyme that catalyzes the rate‐limiting step of citrulline conversion to argininosuccinate in arginine biosynthesis.^[^ ^14^ ^]^ Considering the critical role of arginine and NO in EC function, we speculated that SIRT3, with its known role in protecting EC function, is very likely to be involved in NO production, and might be related to arginine biosynthesis. Therefore, using the endothelium selective Sirt3 knockout mice, we performed a more comprehensive study on the detailed role of SIRT3 in EC function during atherosclerosis and demonstrated that ASS1‐dependent arginine biosynthesis as a target of SIRT3 in EC.

## 2 Results and Discussion

### 2.1 Loss of Endothelial SIRT3 Exacerbates Atherosclerotic Plaque Formation in Mice

**Figure 1:**
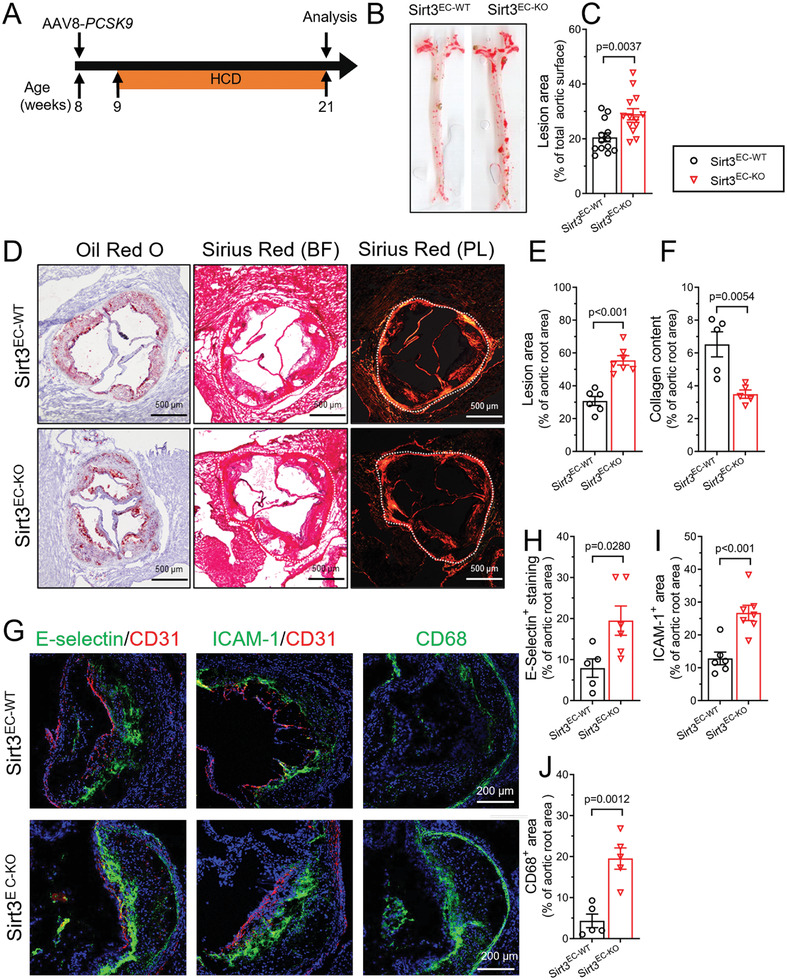
Loss of endothelial SIRT3 promotes plaque deposition and inflammation in HCD‐induced atherosclerosis. A) Schematic experimental outline for atherosclerotic mouse model development. Sirt3^EC‐WT^ and Sirt3^EC‐KO^ mice were injected with AAV8‐mPCSK9 (2.5 × 10^11^ vg per mouse ) via the tail vein and fed with HCD for 12 weeks. Mice were sacrificed at 12 weeks and atherosclerotic plaques were quantified. B,C) Representative images B) and statistical analysis C) of en face Oil Red O positive area of the aorta, expressed as % of total aorta area, *n* = 13 per group. D) Representative images of cross‐sectional Oil Red O staining and cross‐sectional Sirius Red staining of the aortic root. BF: Bright field. PL: polarized light. Scar bar = 500 µm. E,F) Statistical analysis of cross‐sectional Oil Red O‐stained plaque deposition E) and Sirius Red‐stained collagen formation F) in aortic root sections, expressed as % of the total aortic root area. *n* = 5–7 per group. G–J) Representative images G) and statistical analysis H–J) of immunofluorescence staining of E‐Selectin and ICAM‐1 and CD68‐positive macrophages in aortic root sections, expressed as % of the total aortic root area. Scale bar = 200 µm. *n* = 5–7 per group. Data are expressed as mean ± SEM. Statistical significance was calculated by Student's t‐test for comparison between two samples.

**Figure 2:**
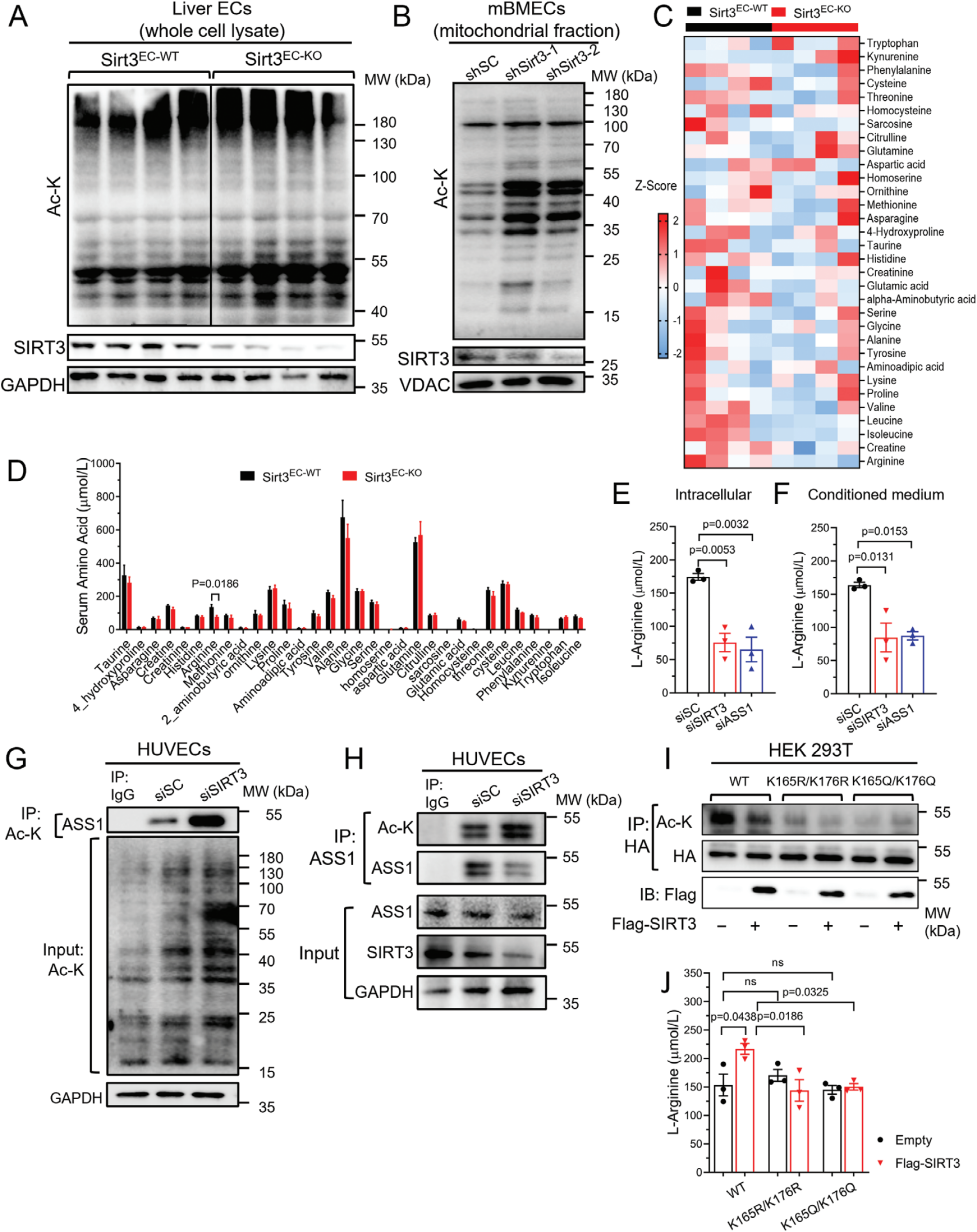
SIRT3 regulates L‐arginine biosynthesis via ASS1 deacetylation in ECs. A) Western blot analysis of the acetylation levels of total protein in ECs isolated from the liver of Sirt3^EC‐WT^ and Sirt3^EC‐KO^ mice. B) Western blot analysis of the acetylated lysine (Ac‐K) levels of mitochondrial protein in mBMECs transfected with shRNA to knock down SIRT3. C) Heat map of serum amino acid enrichment values from Sirt3^EC‐WT^ and Sirt3^EC‐KO^ mice injected with AAV‐mPCSK9 and fed with HCD for 12 weeks. The amino acid concentration was detected by LC‐MS. *n* = 4 per group. D) Serum amino acid profile from Sirt3^EC‐WT^ and Sirt3^EC‐KO^ mice injected with AAV‐mPCSK9 and fed with HCD for 12 weeks. *n* = 4 per group. E,F) L‐Arginine concentration in the intracellular E) and conditioned medium F) from HUVECs transfected with siSC, siSIRT3, or siASS1. G) CO‐IP results of endogenous acetylated lysine were immunoprecipitated in HUVECs transfected with siSC or siSirt3 and the expression of ASS1 was determined by western blotting with an anti‐ASS1 antibody. H) Endogenous ASS1 was immunoprecipitated in HUVECs transfected with siSC or siSIRT3 and its acetylation was determined by western blotting with an anti‐acetylated lysine antibody. I) HA‐tagged WT, K165R/K176R, or K165Q/K176Q mutant ASS1 was transfected into 293T cells together with Flag‐tagged SIRT3 or empty plasmids. Acetylation was determined by western blotting with an anti‐acetyl‐lysine antibody in the immunoprecipitated Flag. J) L‐Arginine concentration in 293T cells transfected with HA‐tagged WT, K165R/K176R, or K165Q/K176Q mutant ASS1 together with Flag‐tagged SIRT3 or empty plasmids. Data are expressed as mean ± SEM. Statistical significance was calculated by Student's t‐test for comparison between two samples, and one‐way ANOVA followed by Tukey's multiple comparisons test for more than two samples.

To investigate the role of endothelium SIRT3 in atherosclerosis, we generated the endothelium‐selective Sirt3 knockout mice (Sirt3^EC‐KO^) and wild‐type mice (Sirt3^EC‐WT^). The knockout efficiency was confirmed in the endothelium of the aorta (Figure S1A–C, Supporting Information) and the magnetic beads sorted CD45^−^CD31^+^ ECs from the liver (Figure 2A), both showing diminished SIRT3 protein expression. We induced experimental atherosclerosis in mice by injecting the adeno‐associated virus to express the proprotein convertase subtilisin/kexin type 9 (AAV‐PCSK9), which increased the expression of *PCSK9* and degrades low‐density lipoprotein receptor (LDLR)  in mouse livers (Figure S2A–B, Supporting Information). To investigate whether EC‐specific deletion of Sirt3 affects atherosclerosis, the AAV‐PCSK9‐injected Sirt3^EC‐WT^ mice and Sirt3^EC‐KO^ mice were fed with high‐cholesterol diet (HCD) for 12 weeks (**Figure** **1**A). The serum total cholesterol level, LDL cholesterol (LDL‐C), and HDL cholesterol (HDL‐C) showed no difference (**Figure** **2**C–E), whereas serum triglyceride levels were slightly but significantly higher in Sirt3^EC‐KO^ mice than in the Sirt3^EC‐WT^ mice (Figure S2F, Supporting Information). Plaque coverage, which was measured by Oil Red O staining on the en face preparation of the aorta, was significantly more in the Sirt3^EC‐KO^ mice, than in the Sirt3^EC‐WT^ mice (Figure 1B,C). Plaque area in the aortic root section was also more in the Sirt3^EC‐KO^ mice (Figure 1D,E). Collagen content within the plaque area, which was measured by Sirius Red staining, showed a marked reduction in the Sirt3^EC‐KO^ mice (Figure 1D,F), suggesting reduced plaque stability. These histological features showed that selective ablation of Sirt3 in the ECs exacerbates the atherosclerotic plaque formation, with unstable plaque characteristics.

Next, to understand whether endothelial SIRT3 is protective against vascular inflammation, we examined the expression of proinflammatory adhesion molecules on plaque ECs. Both E‐Selectin and ICAM‐1 expression were further increased in the intima of the plaque area from Sirt3^EC‐KO^ mice (Figure 1G–I). In addition, the plaque endothelium, which was stained with CD31, also became more discontinuous and punctuated. Macrophage content indicated by CD68 staining, which is a result of EC activation followed by macrophage recruitment and infiltration, also further increased within the plaque area of Sirt3^EC‐KO^ mice (Figure 1G,J). These results suggested that the deletion of endothelial SIRT3 induces a more inflamed EC phenotype with more vascular inflammation in the plaque of Sirt3^EC‐KO^ mice.

### 2.2 Endothelial SIRT3 Regulates L‐Arginine Biosynthesis via ASS1 Deacetylation

To understand how SIRT3 regulates vascular homeostasis, we next aim to explore the target of SIRT3. Primarily functioning as a protein deacetylase in the mitochondria,^[^ ^15^ ^]^ there are several targets of SIRT3 including SOD2 and IDH2 in ECs,^[^ ^9^ ^]^ which have been identified by previous studies. We first examined the acetylation of proteins in several EC types. In ECs isolated from the liver of Sirt3^EC‐KO^ mice, we observed a general increase in acetylated lysine (abbreviated as Ac‐K) in the absence of SIRT3 protein (Figure 2A). In immortalized mouse EC cell line mBMECs after Sirt3 knockdown by stable lentivirus‐shRNA expression, mitochondrial proteins were hyperacetylated (Figure 2B). Similarly, in HUVECs, inhibition of SIRT3 by siRNA (Figure S3A, Supporting Information) also induced protein hyperacetylation, as compared to scramble control (Figure 2G). These results indicated that similar to metabolic tissues, SIRT3 regulates the overall acetylation level of mitochondrial proteins in mouse and human ECs.

To further understand how the alteration of mitochondrial protein acetylation may affect EC metabolism, a targeted high‐throughput amino acid profiling analysis was conducted on serum samples obtained from atherosclerotic Sirt3 ^EC‐WT^ and Sirt3^EC‐KO^ mice using UPLC‐MS/MS (Figure 2C). This metabolomic result showed a significant reduction of L‐arginine concentration in the serum of Sirt3 ^EC‐KO^ mice compared to the wild‐type controls (Figure 2D). Similar trends were validated in the HUVECs with SIRT3 silencing. Both intracellular and secreted levels of L‐arginine were significantly lower in the silenced cells compared to the control group (Figure 2E,F), suggesting that the targets of SIRT3 are likely to be involved in regulating either arginine biosynthesis or arginine catabolism. Notably, reduced arginine availability has long been recognized as a critical factor contributing to endothelial dysfunction and the predisposition to atherosclerotic plaque formation.^[^ ^16^ ^]^ ASS1 is the enzyme responsible for catalyzing the rate‐limiting step in the biosynthesis of arginine from citrulline and was abundant in the mitochondria of ECs (Figure S3B, Supporting Information). Importantly, the silencing of ASS1 in HUVECs led to a reduction in arginine concentration, mirroring the effect observed upon silencing of SIRT3 (Figure 2E,F). These findings collectively suggest a hypothesis that ASS1 may represent a novel and functionally significant target of SIRT3 in ECs due to its regulation of arginine metabolism. Consequently, we further investigated whether ASS1 acetylation status is regulated by SIRT3. In HUVECs with SIRT3 silencing, ASS1 protein was hyperacetylated, which was indicated by co‐IP showing more ASS1 in the immunoprecipitate of Ac‐K from SIRT3 siRNA transfected HUVECs (Figure 2G). Likewise, with SIRT3 silencing in HUVECs, the immunoprecipitate of ASS1 also showed a higher level of Ac‐K, whereas the total expression and localization of ASS1 were unaffected (Figure 2H; Figure S3B, Supporting Information).

Next, to further identify the amino acid residue on ASS1 regulated by acetylation, we selected the previously reported sites K165 and K176 on ASS1. These two residues exhibit high conservation across species, residing within the catalytic site which is crucial for ASS1 activity, and in proximity with the substrates citrulline and aspartate.^[^ ^17^ ^]^ Previous studies also suggested that both K165 and K176 lysine residues can be acetylated by CLOCK protein, resulting in a decrease in ASS1 enzymatic activity.^[^ ^17b^ ^]^ We subsequently examined whether SIRT3 also acts on these lysine residues. HEK293T cells were transfected with pcDNA3.1b‐SIRT3‐FLAG plasmid to overexpress Flag‐SIRT3. Cells were also co‐expressed with HA‐tagged wild‐type ASS1 (WT), or with mutant K165R/K176R or K165Q/K176Q, where lysine (K) was replaced by either arginine (R) or glutamine (Q), both of which are resistant to acetylation. In cells with WT‐ASS1, SIRT3 expression reduced acetylation of HA‐tagged ASS1, while both mutants showed diminished ASS1 acetylation, as indicated by Ac‐K expression in the immunoprecipitate of HA (Figure 2I). Importantly, in the HEK293T cells expressing SIRT3 and WT ASS1, the L‐arginine level was higher, whereas cells expressing both mutant‐ASS1 did not show any increase with SIRT3 expression (Figure 2J). These results demonstrated that SIRT3 regulates ASS1 acetylation and its enzymatic activity in arginine biosynthesis in ECs.

### 2.3 SIRT3 Regulates NO and Redox Status Through L‐Arginine in Endothelial Cells

**Figure 3:**
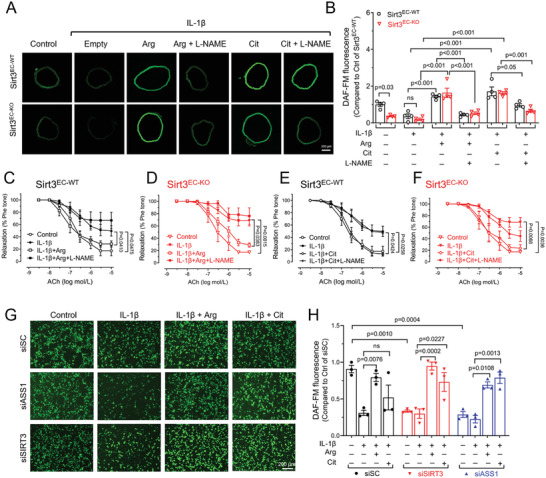
SIRT3 regulates NO production and vascular function via L‐arginine in ECs . A,B) Representative images A) and statistical analysis B) of nitric oxide (NO) production in a cross‐section of the aorta stained with NO indicator DAF‐FM. The aortas were incubated with L‐arginine (Arg, 1 mmol L^−1^), L‐citrulline (Cit, 1 mmol L^−1^), IL‐1β (10 ng mL^−1^), and NOS inhibitor (L‐NAME, 100 µmol L^−1^) for 2 h, alone or in combination. Scale bar = 200 µm. *n* = 4 per group. C,D) The effect of L‐arginine on endothelium‐dependent relaxation (EDR) of aortic segments in response to acetylcholine (ACh) from Sirt3^EC‐WT^ C) and Sirt3^EC‐KO^ D) mice. E,F) The effect of L‐citrulline on EDR of aortic segments in response to ACh from Sirt3^EC‐WT^ E) and Sirt3^EC‐KO^ F) mice. The aorta segments were incubated with Arg (1 mmol L^−1^), Cit (1 mmol L^−1^), L‐NAME (100 µmol L^−1^), and IL‐1β (10 ng mL^−1^), alone or in combination for 2 h. *n* = 5 per group. G‐H) Representative images G) and statistical analysis H) of DAF‐FM fluorescence in HUVECs transfected with siSC, siSIRT3, or siASS1. The HUVECs were pre‐treated with Arg (1 mmol L^−1^), Cit (1 mmol L^−1^), and IL‐1β (10 ng mL^‐1^) for 2 h, alone or in combination. Scale bar = 200 µm. Data are expressed as mean ± SEM. Statistical significance was calculated by one‐way ANOVA followed by Tukey's multiple comparisons test for more than two samples.

Based on the observation of ASS1 and arginine biosynthesis in ECs regulated by SIRT3, we further examined whether the vascular phenotype resulting from SIRT3 loss‐of‐function could be rescued by adding back L‐arginine or the ASS1 substrate L‐citrulline. In the aorta from Sirt3^EC‐KO^ mice, basal levels of NO production, measured by DAF‐FM fluorescence, were significantly reduced. IL‐1β suppressed NO production in both genotypes, whereas both L‐arginine and L‐citrulline increased NO production (**Figure** **3**A,B). In accordance with NO production, endothelial NO‐dependent vasodilation, initially comparable at the basal level between genotypes, exhibited substantial impairment upon IL‐1β stimulation in the aorta, and this impairment was consistent across both genotypes (Figure 3C,D). L‐arginine treatment significantly improved the vasodilatation in both genotypes, although the effect of L‐arginine in IL‐1β treated aorta from Sirt3^EC‐KO^ mice was slightly less than that from Sirt3^EC‐WT^ mice, albeit statistically insignificant (Figure 3D). Similar to L‐arginine, L‐citrulline also improved the vasodilation in both mice impaired by IL‐1β (Figure 3E,F). The effects of L‐NAME to inhibit NO production were similar across all groups indicating that it is NO‐dependent.

To further confirm the observed effects in vitro, DAF fluorescence was also used to measure NO in HUVECs. Silencing of SIRT3 or ASS1 showed a similar effect in inhibiting basal NO production in HUVECs. The impaired NO production induced by IL‐1β was effectively restored by either L‐arginine or L‐citrulline in cells from both siSIRT3 and siASS1 groups (Figure 3G,H). These results indicated a potent effect of IL‐1β to reduce NO production, in addition to its known pro‐inflammatory function. Deletion of SIRT3 reduces NO production, which could be restored by increasing L‐arginine, suggesting that impaired vasodilation and NO production are caused by endogenous L‐arginine synthesis.

**Figure 4:**
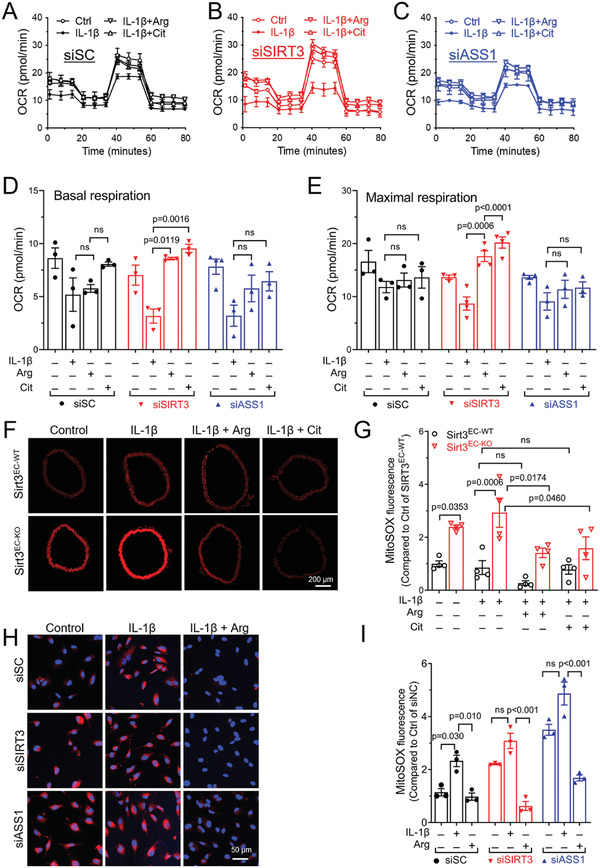
SIRT3 regulates mitochondrial respiratory function and ROS production via L‐arginine in ECs. A–C) Oxygen consumption rate (OCR) was measured by Seahorse XF Cell Mito Stress Test Kit in HUVECs transfected with siSC A), siSIRT3 B), and siASS1 C). The cells were treated with L‐arginine (Arg, 1 mmol L^−1^), L‐citrulline (Cit, 1 mmol L^−1^), and IL‐1β (10 ng mL^−1^) for 16 h, alone or in combination. D–E) Quantification and statistical analysis of basal respiration D), and maximal respiration E) in HUVECs transfected with siSC, siSIRT3, or siASS1. F,G) Representative images F) and statistical analysis G) of mtROS production in aorta rings stained with MitoSOX. The aortas were pre‐treated with Arg (1 mmol L^‐1^), Cit (1 mmol L^‐1^), and IL‐1β (10 ng mL^−1^) for 2 h, alone or in combination. Scale bar = 200 µm. *n* = 4 per group. H,I) Representative images H) and statistical analysis I) of MitoSOX fluorescence in HUVECs transfected with siSC, siSIRT3, or siASS1 The HUVECs were pre‐treated with Arg (1 mmol L^‐1^), Cit (1 mmol L^‐1^), and IL‐1β (10 ng mL^−1^) for 2 h, alone or in combination. Scale bar = 50 µm. Data are expressed as mean ± SEM. Statistical significance was calculated by one‐way ANOVA followed by Tukey's multiple comparisons test for more than two samples.

Arginine deprivation has been implicated in various mitochondrial diseases due to its association with mitochondrial dysfunction.^[^ ^18^ ^]^ Therefore, we assessed mitochondrial function using the seahorse XF method in HUVECs to explore the effect of L‐arginine to rescue mitochondrial dysfunction resulting from SIRT3 inhibition. With stimulation, HUVECs from all the groups exhibited similar basal respiration rates. IL‐1β suppressed both basal and maximal respiration more in SIRT3 or ASS1 silenced cells compared to control (siSC) cells (**Figure** **4**A–C, analysis in Figure 4D,E). The treatment with L‐arginine or L‐citrulline restored respiration impaired by IL‐1β in SIRT3 silenced cells, whereas the effect of either L‐arginine or L‐citrulline in ASS1 silenced cells was insignificant, especially the maximal respiration rate, indicating that increasing L‐arginine biosynthesis is necessary for mitochondrial function.

Impaired mitochondrial function can lead to increased mitochondrial ROS (mitoROS) production. Redox status also affects NO homeostasis in ECs. We used MitoSOX fluorescence to measure mitoROS in ECs and aorta. As expected, the aorta of Sirt3^EC‐KO^ mice showed increased mitoROS production at the basal level, which was further enhanced by IL‐1β (Figure 4F). Both L‐arginine and L‐citrulline suppressed mitoROS in the aorta of Sirt3^EC‐KO^ mice (Figure 4F,G). In HUVECs, silencing of either SIRT3 or ASS1 had a similar effect to increase basal mitoROS level and enhance IL‐1β induced mitoROS overproduction (Figure 4H,I). L‐Arginine reduced mitoROS production in all three groups of HUVECs. These results indicated that SIRT3 deletion sensitizes ECs to IL‐1β induced mitochondrial dysfunction and mitoROS production, which involves ASS1 activity and arginine availability regulated by SIRT3.

### 2.4 Inhibition of SIRT3‐ASS1 Enhances Endothelial Inflammation

**Figure 5:**
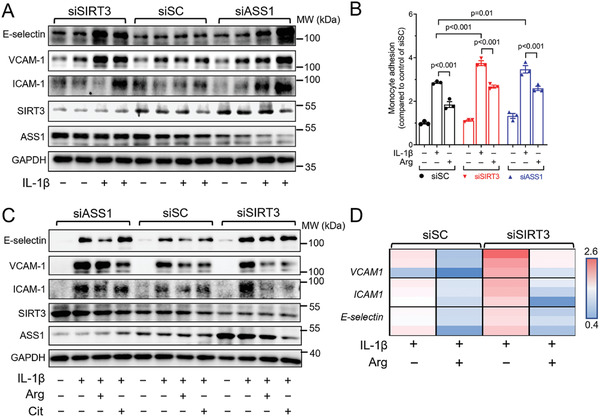
SIRT3 attenuates endothelial inflammation and monocyte adhesion through L‐arginine in ECs. A) Western blot analysis of the endothelial adhesion molecules including E‐Selectin, VCAM‐1, ICAM‐1 in HUVECs transfected with siSC, siSIRT3, and siASS1 stimulated by IL‐1β (10 ng mL^−1^) treatment. B) Statistical analysis of BCECF‐AM‐labeled THP‐1 cells adhering to HUVECs. HUVECs were transfected with siSC, siSIRT3, and siASS1 and treated with L‐arginine (Arg, 1 mmol L^−1^) combined with IL‐1β (10 ng mL^−1^). C) Western blot results of the endothelial adhesion molecules in HUVECs transfected with siSC, siSIRT3, and siASS1. The cells were treated with Arg (1 mmol L^−1^), Cit (1 mmol L^−1^), and IL‐1β (10 ng mL L^−1^), alone or in combination. D) Heatmap showing the mRNA levels of *VCAM‐1, ICAM‐1*, and *E‐Selectin* in HUVECs transfected with siSC or siSIRT3 and treated with Arg (1 mmol L^−1^) combined with IL‐1β (10 ng mL^−1^). Data are expressed as mean ± SEM. Statistical significance was calculated by one‐way ANOVA followed by Tukey's multiple comparisons test for more than two samples.

Endothelial activation is an initial step for the development of atherosclerosis, which also involves the disturbance of NO and redox status. We examined the markers for EC activation and inflammation including adhesion molecules E‐selectin, VCAM‐1, and ICAM‐1, which mediates monocyte adhesion to ECs. Silencing of SIRT3 or ASS1 potentiates the upregulation of all the adhesion molecules induced by IL‐1β (**Figure** **5**A), resulting in more monocyte adhesion, which was measured by THP‐1 cell adhesion on HUVEC monolayer (Figure 5B). In addition, L‐arginine or L‐citrulline suppressed the upregulation of VCAM‐1 and ICAM1 in both control (Figure S4A–D, Supporting Information) and SIRT3 silenced ECs at protein level (Figure 5C), and at mRNA level (Figure 5D, detailed analysis in Figure S4H–K, Supporting Information), while the effect was not present in ASS1 silenced cells (Figure 5C), although functionally L‐arginine was able to attenuate the enhanced THP‐1 cell adhesion induced by IL‐1β (Figure 5B). In addition, the anti‐inflammatory effect of arginine is NO‐dependent, because the L‐arginine‐induced suppression of THP1 adhesion and E‐selectin inhibition were reversed by L‐NAME (Figure S4E–G, Supporting Information). These data suggested that ASS1‐dependent arginine biosynthesis is critical not only for the NO bioavailability but also for the anti‐inflammatory effect of NO in ECs, which is maintained by SIRT3‐dependent deacetylation.

### 2.5 L‐Arginine Supplementation Attenuates Atherosclerosis in SIRT3‐Deficient Mice

**Figure 6:**
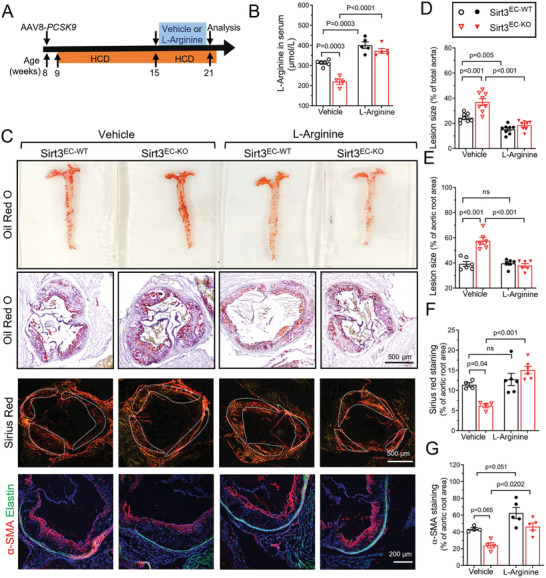
L‐Arginine supplementation attenuates atherosclerosis progression in Sirt3 ^EC‐KO^ mice. A) Schematic experimental outline for the supplementation of L‐arginine in atherosclerotic mice. Sirt3^EC‐WT^ and Sirt3^EC‐KO^ mice were injected with AAV8‐mPCSK9 (2.5 × 10^11^ vg mice^−1^) via tail vein and fed with HCD for 6 weeks, the mice were subsequently randomly divided into two groups, the vehicle (water) group, and L‐arginine (1% in drinking water) group and fed with HCD for another 6 weeks at the same time. B) L‐Arginine concentration in the serum of Sirt3^EC‐WT^ and Sirt3^EC‐KO^ mice supplemented with vehicle or L‐arginine. *n* = 4–6 per group. C) Representative images of en face Oil Red O staining of the aorta (top panels), aortic root cross‐section (middle panels). collagen deposition in frozen aortic root sections indicated by Sirius Red staining (middle panels), Scar bar = 500 µm. Immunofluorescence staining of a‐SMA (bottom panels) (red) in the frozen aortic root area. Scar bar = 200 µm. D,E) Quantification of Oil Red O staining in the aorta enface and aortic root cross‐section. n = 6‐8 per group. F,G) Sirius Red staining F), and immunofluorescence staining of a‐SMA G) in the aortic root cross‐section. *n* = 4–6 per group. Data are expressed as mean ± SEM. Statistical significance was calculated by one‐way ANOVA followed by Tukey's multiple comparisons test for more than two samples.

Based on the evidence from HUVECs and isolated mouse aorta, we sought to examine the effect of restoring arginine level in vivo to rescue the SIRT3 deletion‐induced vascular pathology in atherosclerosis. Both Sirt3^EC‐WT^ and Sirt3^EC‐KO^ mice were injected with AAV8‐PCSK9 via tail vein injection and fed with HCD to induce atherosclerosis. After 6 weeks, 1% L‐arginine in drinking water was given to the mice on HCD for an additional 6 weeks (**Figure** **6**A). L‐Arginine treatment increased circulating L‐arginine concentration in both Sirt3^EC‐WT^ and Sirt3^EC‐KO^ mice (Figure 6B). Of note, L‐arginine treatment did not affect body weight, heart, and cholesterol, although the triglyceride level was higher in the Sirt3^EC‐KO^ mice (Figure S5A–E, Supporting Information). Oil Red O staining for the lipid content of plaque in both the en face preparation of aorta (Figure 6C,D) and the aortic root area analysis (Figure 6C,E) showed ≈50% reduction of plaque area in Sirt3^EC‐KO^ mice, whereas the effect in Sirt3^EC‐WT^ mice was less, although significant in the total plaque area of aorta, but insignificant in the aortic root (Figure 6D,E). Plaque stability was measured by collagen content (Sirius Red) and α‐SMA as a smooth muscle marker for the fibrous cap. The reductions of collagen and α‐SMA in the plaque area of the Sirt3^EC‐KO^ mice were rescued by L‐arginine treatment (Figure 6C,F,G). Notably, L‐citrulline supplementation has no effect on plaque deposition in the aorta from both Sirt3^EC‐WT^ and Sirt3^EC‐KO^ mice (Figure S6A–C, Supporting Information).

**Figure 7:**
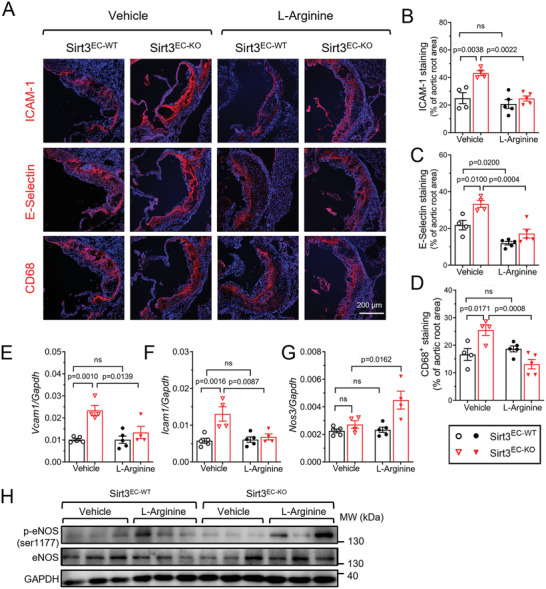
L‐Arginine supplementation attenuated vascular inflammation and macrophage infiltration in Sirt3^EC‐KO^ mice. A–D) Representative images A) and statistical analysis of ICAM‐1 B), E‐Selectin C), and CD68 D) in aortic root cross sections. Scar bar = 200 µm, *n* = 4–5 per group. E–G) qPCR results of mRNA levels of *Vcam‐1* E), *Icam‐1* F), and *Nos3* G) in the isolated aorta from Sirt3^EC‐WT^ and Sirt3^EC‐KO^ mice supplemented with vehicle or L‐arginine. *n* = 3 per group. H) Western blot analysis of the expression of eNOS and p‐eNOS(s1177) in the isolated aorta from Sirt3^EC‐WT^ and Sirt3^EC‐KO^ mice supplemented with vehicle or L‐arginine. *n* = 3 per group. Data are expressed as mean ± SEM. Statistical significance was calculated by one‐way ANOVA followed by Tukey's multiple comparisons test for more than two samples.

We also examined the inflammatory markers in the mice following L‐arginine treatment. L‐Arginine attenuated the upregulation of ICAM‐1, E‐selectin in the plaque area (**Figure** **7**A–C), and the *Icam1* and *Vcam1* expression at mRNA level in the thoracic aorta (Figure 7E,F) in the Sirt3^EC‐KO^ mice, but not Sirt3^EC‐WT^ mice. Macrophage infiltration as a result of EC activation, measured by CD68 immunofluorescence, was also suppressed by L‐arginine treatment in the Sirt3^EC‐KO^ mice (Figure 7A,D). We also observed a notable increase of *Nos3 e*xpression (mRNA in Figure 7G, and protein in Figure 7H), in the Sirt3^EC‐WT^ mice after L‐arginine treatment, without much change of eNOS phosphorylation (Figure 7H), suggesting that arginine level and possibly ASS1 activity might help to stabilize eNOS in ECs.^[^ ^19^ ^]^ To summarize, these results indicated that the enhanced plaque formation, plaque instability, and exacerbated vascular inflammation due to endothelial SIRT3 deletion is probably due to impaired ASS1‐mediated arginine synthesis in the endothelium.

**Figure 8:**
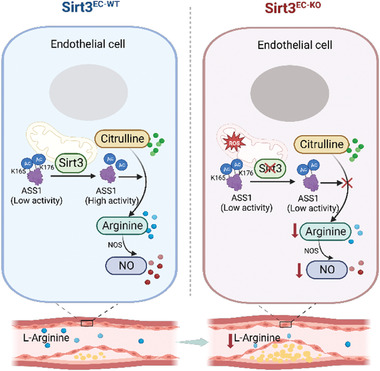
Hypothesis model for the role of endothelial Sirt3 in atherosclerosis. Deletion of endothelial SIRT3 leads to the hyperacetylation of ASS1, which, in turn, blocks the L‐arginine synthesis pathway. This reduction in L‐arginine levels subsequently leads to a decreased production of nitric oxide (NO), resulting in endothelial dysfunction and the progression of atherosclerotic plaques. The graphic was generated using BioRender.com, and a publication license has been secured (License number: AE266IGSTE).

## 3 Conclusion

In the present study, we demonstrated that the intrinsic L‐arginine biosynthesis mediated by ASS1 in ECs plays an important role in vascular homeostasis. ASS1 activity is largely controlled by SIRT3‐dependent deacetylation. Without SIRT3, impaired ASS1 activity and L‐arginine deficiency are critical factors contributing to mitochondrial dysfunction, disturbance of NO and redox status, endothelial inflammation, and enhanced atherosclerosis induced by SIRT3 deletion in mice (**Figure** **8**).

SIRT3 is an NAD^+^‐dependent protein deacetylase that exhibits decreased expression during various types of vascular pathologies including vascular senescence, hypertension, and atherosclerosis.^[^ ^20^ ^]^ The protective role of SIRT3 in the endothelium has been extensively studied, mainly on oxidative stress and vascular inflammation.^[^ ^21^ ^]^ For instance, SIRT3 protects EC against oxidative stress through the deacetylation of SOD2, which was demonstrated using the global Sirt3^−/−^ and Sirt3 overexpression mice.^[^ ^9a^ ^]^ SIRT3 also protects EC phenotype through the deacetylation of Foxo3a and its downstream catalase to reduce oxidative stress, as evidenced in global Sirt3^−/−^ and EC‐selective Sirt3 overexpression mice.^[^ ^22^ ^]^ Regarding vascular function, our observation was similar to the previous study showing mild endothelial dysfunction in the Sirt3^−/−^ mice fed with HCD.^[^ ^23^ ^]^ Our present data using either isolated aorta from Sirt3^EC‐KO^ mice or SIRT3 silenced HUVECs are in agreement with these previous studies. In general, SIRT3 inhibition causes endothelial dysfunction and worsens endothelial inflammation in the presence of proinflammatory stimuli.

In a previous study by Winnick et al., the role of SIRT3 during atherosclerotic plaque formation was investigated by using global Sirt3^−/−^ mice on the Ldlr^−/−^ background fed with the same diet as the one used in our study. The study did not show any significant differences in plaque formation or pathology between the Sirt3^−/−^ mice and the wild‐type mice on Ldlr^−/−^ background.^[^ ^24^ ^]^ Meanwhile, these mice showed similar metabolic phenotypes such as more weight gain, compared to C57/BL6 mice without Ldlr^−/−^ on a high‐fat diet.^[^ ^24^ ^]^ There is no report of Sirt3^−/−^ mice on the Apoe^−/−^ background for atherosclerosis study. In our study using the AAV to overexpress PCSK9 and reduce LDLR expression, we expect a similar lipid profile as the Ldlr^−/−^ mice. Therefore, we reasoned that the differences could be attributed to the contribution of other cells or organs, beyond those involved in lipid and cholesterol metabolism. In addition to vascular cells, myeloid cells are also present in the plaque. While there is no report on the role of SIRT3 in myeloid cells during atherosclerosis using cell‐selective knockout mice, prior research involving Sirt3^−/−^ mice showed increased neutrophil extracellular trap and plasma tissue factor activity in non‐atherosclerotic thrombosis model.^[^ ^25^ ^]^ Further studies using myeloid selective Sirt3 knockout mice have shown that Sirt3 deletion increases inflammasome activity, which relates to PDHA1 deacetylation in the perivascular macrophages.^[^ ^26^ ^]^ Both studies agree with a more inflammatory phenotype of myeloid cells with SIRT3 inhibition, similar to its effect in ECs. However, the role of SIRT3 in myeloid cells or other immune cells in the context of atherosclerosis remains largely unknown. Although previous studies have suggested that upregulating or activating SIRT3 improves endothelial function and attenuates atherosclerosis in mice, in our study, we have provided more direct evidence to support this claim, that endothelial SIRT3 plays an important role in maintaining vascular homeostasis. This is exemplified by the increased plaque size and more unstable plaque pathology resulting from the deletion of endothelial Sirt3.

In addition to endothelial activation and inflammation, the reduction in atherosclerotic plaque size observed in our study could be due to a lipid‐lowering effect. SIRT3 is well‐known for suppressing lipogenesis and stimulating fatty acid oxidation in the liver.^[^ ^15^, ^27^ ^]^ Previous studies have demonstrated that Sirt3^−/−^ mice on a high‐fat diet experience more weight gain and exhibit higher levels of triglyceride, cholesterol, and LDL‐cholesterol in the liver.^[^ ^27^, ^28^ ^]^ In our study, we observed a mild but significant increase in blood triglyceride levels in HCD‐fed Sirt3^EC‐KO^ mice. This may be related to the moderate upregulation of vascular inflammation in the liver ECs, which may facilitate an increase in triglyceride transport and release. However, we found that liver histology and lipid accumulation were similar between Sirt3^EC‐KO^ mice and Sirt3^EC‐KO^ mice (data not shown). Therefore, the changes in triglyceride are unlikely to contribute to the observed differences in plaque size and pathology in our study.

While the function of SIRT3 in ECs is well‐established, many of its direct targets in the context of EC function have yet to be fully revealed. In our study, we drew inspiration from previous research conducted on the acetylome of liver proteins under conditions of a high‐fat diet or Sirt3 deletion.^[^ ^15^, ^28^ ^]^ Specifically, we focused on urea cycle enzymes which are also involved in NO production. We observed a lower L‐arginine concentration in the SIRT3^EC‐KO^ mice, which suggests that enzymes involved in L‐arginine biosynthesis may be affected by the loss of SIRT3 expression. Notably, previous studies have reported that Sirt3^−/−^ mice have lower argininosuccinate levels but normal arginine levels in the liver, which is largely due to the impairment of Ornithine carbamoyltransferase function.^[^ ^13^ ^]^ Furthermore, it has been observed that the rate‐limiting enzyme in arginine biosynthesis, ASS1, is hyperacetylated in the liver of Sirt3^−/−^ mice.^[^ ^15b^ ^]^ Motivated by this, we investigated the role of ASS1 as a potential target of SIRT3 in ECs. Through our experiments using HUVECs, we found that the silencing of SIRT3 resulted in hyperacetylated ASS1, which subsequently led to lower arginine biosynthesis in these cells. Further experiments focusing on the known lysine residues K165 and K176^[^ ^17b^ ^]^ (both known to be regulated by SIRT3 and involved in the enzymatic activity of ASS1), corroborated these findings. Taken together with the in vivo measurements of arginine concentration, these results suggest that ECs are primarily responsible for the de novo synthesis of L‐arginine, which is regulated by SIRT3‐ASS1.

We then aimed to explore the involvement of ASS1 and L‐arginine in maintaining vascular homeostasis. ASS1 and argininosuccinate lyase (ASL) are both urea cycle enzymes and deficiency in ASS1 and ASL are genetic disorders that can lead to hyperammonemia in humans. Despite this, only a limited number of studies have examined the involvement of ASS1 and ASL in endothelial function. Interestingly, inhibition of either ASS1^[^ ^29^ ^]^ or ASL^[^ ^30^ ^]^ in ECs has been shown to result in decreased NO production, endothelial dysfunction, and vascular inflammation. Endothelial selective ASL knockout mice have been found to exhibit reduced NO production and higher blood pressure. These findings suggested that ECs represent a crucial source for de novo arginine biosynthesis, and unless arginine is supplemented from a dietary source, decreased urea cycle enzyme activity may result in arginine deficiency and impaired NO production. Several previous studies have demonstrated the beneficial role of increasing NO, and supplementation of arginine in improving endothelial dysfunction, slowing the progression of atherosclerosis, which may be due to the effect of arginine on upregulation of eNOS.^[^ ^31^ ^]^ In addition, clinical studies have already provided evidence for the beneficial effects of arginine supplements in various diseases to inhibit inflammation,^[^ ^32^ ^]^ improve glucose metabolism,^[^ ^33^ ^]^ and reduce cardiovascular risk factors.^[^ ^34^ ^]^ These findings further support the importance of de novo arginine biosynthesis for vascular and metabolic homeostasis. Consistent with this, our study demonstrated that replenishing arginine rescues NO production, redox balance, EC activation and inflammation in both SIRT3 silenced HUVECs, and in SIRT3^EC‐KO^ mice, indicating that the worsened vascular pathology observed with SIRT3 inhibition is primarily due to the impairment of ASS1 and arginine biosynthesis.

There are some limitations of our study. We showed the effect of L‐citrulline in vitro in improving NO production, improving vasodilatation, and attenuating vascular oxidative stress in SIRT3 or ASS1 silenced HUVECs, which was similar although not identical to L‐arginine. This effect could be due to the overall increased activity of arginine biosynthesis, and also possibly an inhibitory effect of arginine degradation to ornithine through enzymes such as arginase.^[^ ^35^ ^]^ In fact, previous studies showed that the bioavailability of oral administration arginine is limited due to extensive metabolism by arginase in the gut wall and liver.^[^ ^36^ ^]^ Furthermore, the effects of arginine and urea cycle enzymes involved in lipid and cholesterol metabolism are also undetermined and may require further attention. In summary, our study has provided new insights into the role of endothelial SIRT3 in maintaining vascular homeostasis and preventing atherosclerosis through SIRT3‐mediated deacetylation of ASS1, which is responsible for de novo arginine biosynthesis. This highlights the importance of arginine biosynthesis and urea cycle enzymes in endothelial function, particularly in the context of cardiovascular disease. Further clinical investigations could be useful to fully explore the implications of these findings in clinical settings.

## 4 Experimental Section

### Animals

The Sirt3 floxed mice (Sirt3tm1.*^1Auw^ or Sirt3^L2^*, generously provided by Johan Auwerx Lab) were crossed with the endothelium‐selective Cdh5‐cre transgenic mice (*B6;129^−Tg(Cdh5‐cre)1Spe/J^*, obtained from Jackson lab) to generate endothelial selective deletion of Sirt3 as Sirt3f/f; Cdh5Cre/+ (Sirt3^EC‐KO^) in mice, and their wild type littermates Sirt3f/f (Sirt3^EC‐WT^) as controls. The mice were housed at a constant temperature (22 ± 1°C) with a 12 h light/dark cycle and had free access to water and standard chow unless otherwise specified. All experiments were carried out on littermates and randomly assigned to the experimental groups. Animal studies were conducted under the approval of the Hong Kong Department of Health and in accordance with the guidelines of the Laboratory Animal Experimentation Ethical Committee of the Chinese University of Hong Kong (AEEC: 19‐125‐GRF). All procedures were performed in accordance with the NIH Guide for the Care and Use of Laboratory Animals.

### Experimental Atherosclerosis in Mice

Male mice of each genotype received a single injection of adeno‐associated viruses AAV8‐mPCSK9 (pAV‐HCRApoE‐hAAT‐mPCSK9, Vigene Biosciences, China, 2.5 × 10^11^ vg mouse^−1^) at 8 weeks of age. Subsequently, the mice were fed on the high‐cholesterol diet (HCD, D12336, Research Diets Inc., NJ, USA) for 12 weeks to induce atherosclerosis. After 6 weeks of HCD feeding, some mice were then randomly assigned to two groups either supplemented with L‐arginine (1% in drinking water) or vehicle (water only) for another 6 weeks, while kept on HCD.

### Histological Analyses

The mice were anesthetized by intraperitoneal injection of 80 mg kg^−1^  ketamine and 10 mg kg^−1^ xylazine. The heart was punctured for perfusion with heparin‐containing saline to wash out the blood from the aorta. Hearts were embedded in OCT compound and serially sectioned at 10 µm intervals, starting from the first appearance of three valves of the aortic root. For each mouse, the section with the largest aortic root area and three visible valves was used for analysis. Picrosirius red staining was used to examine collagen deposition in atherosclerotic lesions. Images were taken at 4× magnification under both bright field and polarized light. Oil Red O staining was conducted to detect lipid deposition in both the en‐face preparation of the aorta and the aortic root sections. For en‐face preparation, aortas were dissected and opened longitudinally from the heart to the bifurcation of iliac arteries and pinned out on a silicone plate to evaluate plaque coverage. Both the aortic root sections and aortas were fixed in 4% paraformaldehyde, stained with freshly prepared Oil Red O working solution, washed with ddH_2_O, and mounted in an aqueous mounting medium. Images of the aorta were captured with a Leica dissection microscope. Histological images were captured using a Nikon Ni‐U Eclipse upright microscope. Images were quantitated using ImageJ (National Institutes of Health, Bethesda, MD, USA).

### Immunofluorescence Staining

Cryosections of tissues or cells on coverslips were fixed with 4% paraformaldehyde and then blocked with 5% normal goat or donkey serum for 2 h at room temperature. The sections were subsequently incubated with primary antibodies at a 1:200 dilution overnight at 4 °C, then with appropriate fluorescence‐conjugated secondary antibodies (ThermoFisher, Waltham, MA, USA) at a 1:500 dilution for 2 h at room temperature in the dark. The nuclei were stained with Hoechst 33 342 (ThermoFisher, Waltham, MA, USA) for 15 min at room temperature and then mounted in a fluorescence mounting medium (Electron Microscopy, 17985‐10, Hatfield, PA, USA). Confocal images were obtained using the Olympus FV1200 microscope and ImageJ software was used to quantify the positive fluorescence signal in the area of interest. The list of antibodies used in this study is provided in Table S1 (Supporting Information).

### Serum Chemistry

The concentration of total cholesterol and low‐density lipoprotein cholesterol (LDL‐C) were measured in the serum by colorimetric method (Elabsciences, China) according to the manufacturer's instructions. Triglyceride in the serum was measured by colorimetric method (EKF stanbio, Barleben, Germany) according to the manufacturer's instructions.

### LC‐MS Analysis for Metabolites

Amino acids concentration was measured in mouse serum using a quantitative ultra‐performance liquid chromatography‐tandem mass spectrometry (UPLC‐MS/MS) platform (Acquity UPLC‐Xevo TQ‐S; Waters Corp. Milford, MA, USA. All chromatographic separations were performed with a Cortex UPLC C18 Column VanGuard pre‐column (1.6 × 5 mm) and analytical column (2.1 × 50 mm). The elution solvents were water with 0.2% formic acid (A) and acetonitrile with 0.1% formic acid (B). The flow rate was 500 µl min^−1^ with the following mobile phase gradient: 0–0.5 min (1% B), 0.5–2 min (1%–10% B), 2–2.5 min (10%–15% B), 2.5–4 min (15%–20%B), 4–4.5 min (20%–99%B), 4.5–5 min (99% B), 5–7 min (1% B). The raw data generated by UPLC‐MS/MS were then processed using the QuanMET software (v1.0, Metabo‐Profile, Shanghai, China) to perform peak integration, calibration, and quantitation for each amino acid. L‐arginine concentration in the cells and conditioned cell culture medium was measured by the L‐arginine measurement kit (ab241028, Abcam, MA, USA) according to the manufacturer's instructions.

### Vascular Reactivity

Thoracic aortas were dissected in oxygenated cold Krebs solution, and the adjacent connective tissue and adipose tissue surrounding the aortas were carefully removed. The aortas were then cut into 1.5 mm long segments. Some aortic segments were incubated with various drugs in Dulbecco's Modified Eagle's Media with low glucose (DMEM; Life Technologies) containing 10% fetal bovine serum, and 1% Antibiotic‐Antimycotic. The aorta segments were mounted in a wire myograph system (Danish Myo Technology, Aarhus, Denmark) in the Krebs solution containing (mmol L^‐1^, pH 7.4) 119 NaCl, 4.7 KCl, 2.5 CaCl_2_, 1 MgCl_2_, 25 NaHCO_3_, 1.2 KH_2_PO_4_, and 11.1 D‐glucose. The segments were stretched to a baseline tension at 3 mN and allowed to equilibrate for 60 min at 37 °C. Vessel viability was assessed by the response to 60 mmol L^‐1^ KCl. Endothelial‐dependent relaxation (EDR) was measured by the concentration‐dependent response to cumulated concentrations of acetylcholine (Ach, 3 nmol L^−1^ to 10 mmol L^−1^) in phenylephrine (Phe, 3 µmol L^−1^) precontracted segments. The endothelium‐independent relaxation in response to NO donor sodium nitroprusside (SNP) was also conducted after 30 min of incubation with L‐NAME (100 µmol L^−1^) to inhibit NO synthase.

### NO and ROS Detection

The thoracic aorta was cut into 2–3 mm rings and subjected to drug incubation before being frozen in the OCT compound. Cryosections of the aorta or the adherent cells on the coverslip were incubated in HBSS buffer containing DAF‐FM Diacetate (Invitrogen, D23844, Waltham, MA, USA) at 10 µmol L^−1^ for 20 min in the dark at 37 °C to detect the NO production. Mitochondrial O_2_^•−^ generation was detected using MitoSOX Red indicator (Invitrogen, M36008, Waltham, MA, USA) at 5 µmol L^−1^ for 20 min at 37 °C in HBSS. Fluorescence intensity was captured using an Olympus FV1200 confocal microscope, and the mean fluorescence intensity (MFI) was quantified using ImageJ software.

### Seahorse Test for Mitochondrial Metabolism

Mitochondrial oxygen consumption rate (OCR) of HUVECs was evaluated by using Seahorse XF96 Analyzer (Agilent Technologies, Santa Clara, CA USA) according to the manufacturer's protocol. HUVECs (8000 cells/well) were pre‐treated with or without pharmacological drugs. On the day of the metabolic flux analysis, the cells were equilibrated in XF Base medium (Agilent Technologies, 102353‐100) supplemented with 2 mmol L^‐1^ Glutamax, 1 mmol L^−1^ sodium pyruvate, and 25 mmol L^−1^ glucose and incubated for 1 h in a 37 °C incubator without CO_2_. Following the establishment of a baseline OCR reading, the following mitochondrial respiratory chain protein pharmacological manipulators were injected in the following order: i) oligomycin 1 µmol L^−1^) as an ATP synthase inhibitor; ii) carbonyl cyanide‐4‐(trifluoromethoxy) phenyl hydrazone (FCCP) (1.5 µmol L^‐1^) as a mitochondrial uncoupler; and iii) rotenone (1 µmol L^−1^) as a complex I inhibitor. The Seahorse XF‐96 software was used to record and calculate OCR.

### Cell Culture and Cell Transfection

Mouse brain microvascular endothelial cells (mBMECs, from Angio‐Proteomie) in a 50%–70% confluency were infected with lentivirus carrying three short hairpin RNA (shRNA) sequences targeting mouse Sirt3 or a scramble lentivirus as a native control and selected with 1 ug mL^−1^ puromycin for stable expression. The sequences of shRNA (Hanbio Biotechnology, Shanghai) are listed in Table S3 (Supporting Information):

Human umbilical vein endothelial cells (HUVECs, Lonza, Visp, Switzerland) within passages 6–8 were transfected with siRNA targeting human SIRT3, ASS1, or a universal scrambled negative control siRNA (GenePharma, Suzhou, China) using Lipofectamine RNAiMAX Transfection Reagent (ThermoFisher, Waltham, MA, USA) according to the manufacturer's instructions. The knockdown efficiency was evaluated within 48–72 h post‐transfection. The sequences of shRNA (Hanbio Biotechnology, Shanghai) are listed in Table S3 (Supporting Information).

HEK293T cells (ATCC) in a 50%–70% confluency were transfected with plasmids using Lipofectamine 3000 Transfection Reagent (ThermoFisher, Waltham, MA, USA) following the manufacturer's instructions. Plasmids used in this study include pcDNA3.1b‐Sirt3‐FLAG, pcDNA3.1b‐ASS1(WT)‐HA, pcDNA3.1b‐ASS1(K165R/K176R)‐HA, and pcDNA3.1b‐ASS1(K165Q/K176Q)‐HA (WZ Biosciences Inc, China).

THP1 cells (ATCC) were cultured in RPMI‐1640 Medium supplemented with 0.05 mmol L^−1^ 2‐mercaptoethanol, 10% FBS, and 1% antibiotic‐antimycotic solution.

### Monocyte Adhesion

HUVECs were cultured in 12‐well plates and reached confluence. The THP1 cells were labeled with 5 µmol L^−1^ BCECF‐AM (ThermoFisher, Waltham, MA, USA) for 30 min in the dark at 37 °C and added to HUVECs containing well and incubated for 1 h. Subsequently, the cells were washed with HBSS three times to remove the non‐adherent cells and the adherent monocytes were imaged using a fluorescence microscope (EVOS M5000, ThermoFisher).

### Quantitative Real‐Time PCR

Cells or tissues were lysed in RNAiso plus (TaKaRa) for total RNA extraction and 1 µg RNA was used to reverse‐transcribe into cDNA using PrimeScript RT Master Mix (TaKaRa, RR036A, Beijing, China) according to the manufacturer's instructions. mRNA levels of target genes were determined by quantitative RT‐PCR with TB Green Premix Ex Taq (Takara, RR420A, Beijing, China) on the ViiA real‐time PCR system (Applied Biosystems, Waltham, MA, USA). All Primer sequences are listed in Table S2 (Supporting Information).

### Western Blotting and Co‐Immunoprecipitation

Cell or tissue was homogenized or lysed in ice‐cold RIPA buffer with a Protease inhibitor cocktail and phosphatase inhibitor cocktail (both from Roche, Basel, Switzerland). Mitochondrial proteins were obtained by the protocols as previously described.^[^ ^37^ ^]^ Protein concentration was evaluated by the BCA Protein Assay Kit (Thermo Fisher, 23 225) according to the manufacturer's protocol. The same amount of samples were mixed with 4X loading buffer with 5% β‐mercaptoethanol and denatured at 95 °C for 5 min. Denatured protein samples were loaded and separated in SDS‐PAGE gels and transferred onto the PVDF membrane, the membrane was blocked with 5% BSA and incubated with primary antibody overnight at 4 °C. The membrane was washed with TBST and incubated with goat anti‐rabbit or anti‐mouse IgG horseradish peroxidase secondary antibody at a dilution of 1:2000 at room temperature. Band intensity was detected by reacting with Western Chemiluminescence HRP Substrate (ECL, EMD Millipore) reagent and exposed using BioRad ChemDoc MP Imaging System (Bio‐Rad, California, USA). All antibodies are listed in Table S1 (Supporting Information).

For co‐immunoprecipitation, cells or tissues were lysed in IP lysis buffer (P0013J, Beyotime, China). The clear supernatant was then incubated with different antibodies at the recommended dilution for immunoprecipitation overnight at 4 °C. The mixture was then added to sepharose beads (GE Healthcare) and incubated under rotary agitation at 4 °C for 4 h. The bead pellet was washed in lysis buffer three times and resuspended in Laemmli buffer (2% SDS, 50 mmol L^−1^ Tris‐HCl, pH 6.8, 10% glycerol, 5% β‐mercaptoethanol, 0.0005% Bromophenol Blue) and denature at 95 °C for 5 min to dissociate immunoprecipitants from the beads. All antibodies are listed in Table S1 (Supporting Information).

### Statistical Analysis

All of the data were presented as means ± SEM and the numbers of independent experiments were indicated. Student's unpaired t‐test was used for the comparison between the two groups. One‐way ANOVA with Tukey's post‐test was used to compare differences between multiple experimental groups. All data were analyzed using GraphPad Prism 9. *p* < 0.05, *p* < 0.01, and *p* < 0.001 was considered statistically significant.

## Conflict of Interest

The authors declare no conflict of interest.

## Author Contributions

X.C. and X.Y.T. designed and initiated the study. X.C. performed and analyzed most of the experiments. V.W.Y.W., Y.H., H.H., and Y.W. participated in animal studies. A.P.S.K. and K.O.L. provided critical reagents and reviewed the manuscript. X.C., X.Y.T. wrote the manuscript. X.Y.T. analyzed and interpreted the experiments, and supervised the research. All authors discussed the results and approved the manuscript.

## Supporting information

Supporting Information

## Data Availability

The data presented in this study are available in this manuscript and in its Supplementary material online.
