# Supplementary material for: Role of Argininosuccinate Synthase 1 ‐Dependent L‐Arginine Biosynthesis in the Protective Effect of Endothelial Sirtuin 3 Against Atherosclerosis
Source: Adv Sci (Weinh). 2024 Jan 17;11(12):2307256. doi: 10.1002/advs.202307256 (PMC10966544; doi:10.1002/advs.202307256)
Supplement: Supplementary file 1 — Supporting Information [file ADVS-11-2307256-s001.pdf]

ADVANCED SCIENCE
Open Access

Supporting Information

for *Adv. Sci.*, DOI 10.1002/advs.202307256

Role of Argininosuccinate Synthase 1 -Dependent L-Arginine Biosynthesis in the Protective Effect of Endothelial Sirtuin 3 Against Atherosclerosis

*Xiaoyun Cao, Vivian Wei Yan Wu, Yumeng Han, Huiling Hong, Yalan Wu, Alice Pik Shan Kong, Kathy O Lui and Xiao Yu Tian**

Supporting Information

Role of Argininosuccinate Synthase 1 -Dependent L-Arginine Biosynthesis in the Protective Effect of Endothelial Sirtuin 3 Against Atherosclerosis.

Xiaoyun Cao, Vivian Wei Yan Wu, Yumeng Han, Huiling Hong, Yalan Wu, Alice Pik Shan Kong, Kathy O Lui, Xiao Yu Tian[*]

Supporting Information includes:

Supplementary Tables S1-S3

Supplementary Figures S1-S6

Table S1. List of antibodies.

Target antigen	Source	Catalog	Working concentration
mouse anti-GAPDH loading control antibody	Thermofisher	MA5-15738	1:1000
Recombinant Anti-VCAM1 antibody	Abcam	ab134047	1:1000
E-Selectin Antibody	Santa Cruz	sc-137054	1:200
ICAM-1 Monoclonal Antibody	Santa Cruz	sc-107	1:200
SIRT3 Antibody (C-term)	abcepta	AP6242a	1:1000
Rabbit monoclonal [EPR12398] to ASS1	abcam	ab170952	1:1000
Purified Mouse Anti-eNOS/NOS Type III	BD	610297	1:1000
Purified Mouse Anti-eNOS (pS1177)	BD	612392	1:1000
HA-Tag (F-7): sc-7392	Santa Cruz	sc-7392	1:1000
Flag-Tag Antibody	Affinity	T0003	1:1000
Pan anti-acetyllysine rabbit antibody	PTM Biolabs	PTM-105	1:500
CD68	Abcam	ab31630	1:200
Anti-alpha smooth muscle Actin	Abcam	ab32575	1:500
CD144 (VE-cadherin) monoclonal antibody	Invitrogen	14-1441-82	1:200
Anti-CD31 antibody	Abcam	ab28364	1:500

Table S2. List of primer sequences.

Genes	Forward sequence (5' to 3')	Reverse sequence (5' to 3')
Human GAPDH	TGTGGGCATCAATGGATTTGG	ACACCATGTATTCCGGGTCAAT
Human VCAM-1	CAGTAAGGCAGGCTGTAAAAGA	TGGAGCTGGTAGACCCTCG
Human ICAM-1	TTGGGCATAGAGACCCCGTT	GCACATTGCTCAGTTCATACACC
Human E-SELECTIN	TGTGGGTCTGGGTAGGAACC	AGCTGTGTAGCATAGGGCAAG
Human SIRT3	GACATTCGGGCTGACGTGAT	ACCACATGCAGCAAGAACCTC
Mouse Gapdh	ATGGTGAAGGTCGGTGTGAA	GAGGTCAATGAAGGGGTCGT
Mouse Icam1	AAACCAGACCCTGGAACTGCAC	GCCTGGCATTTCAGAGTCTGCT
Mouse Vcam1	ACAGACAGTCCCCTCAATGG	TCCTCAAAACCCACAGAGCT
Mouse Nos3	TCAGCCATCACAGTGTTCCC	ATAGCCCGCATAGCGTATCAG

Table S3. List of shRNA and siRNA sequences.

Genes	sequence (5' to 3')
scrambled negative shRNA	TTCTCCGAACGT GTCACGTAA
sh-Sirt3-1	CCCGTACCCTGAAGCCATCTTTGAA
sh-Sirt3-2	TCGCTTTGGCAGATCTGCTACTCAT
negative control siRNA	UUCUCCGAACGUGUCACGUTT
siSIRT3	CCAGCAUGAAAUACAUUUA
siASS1	GCAAUGACCUGAUGGAGUA

Supplementary Figures

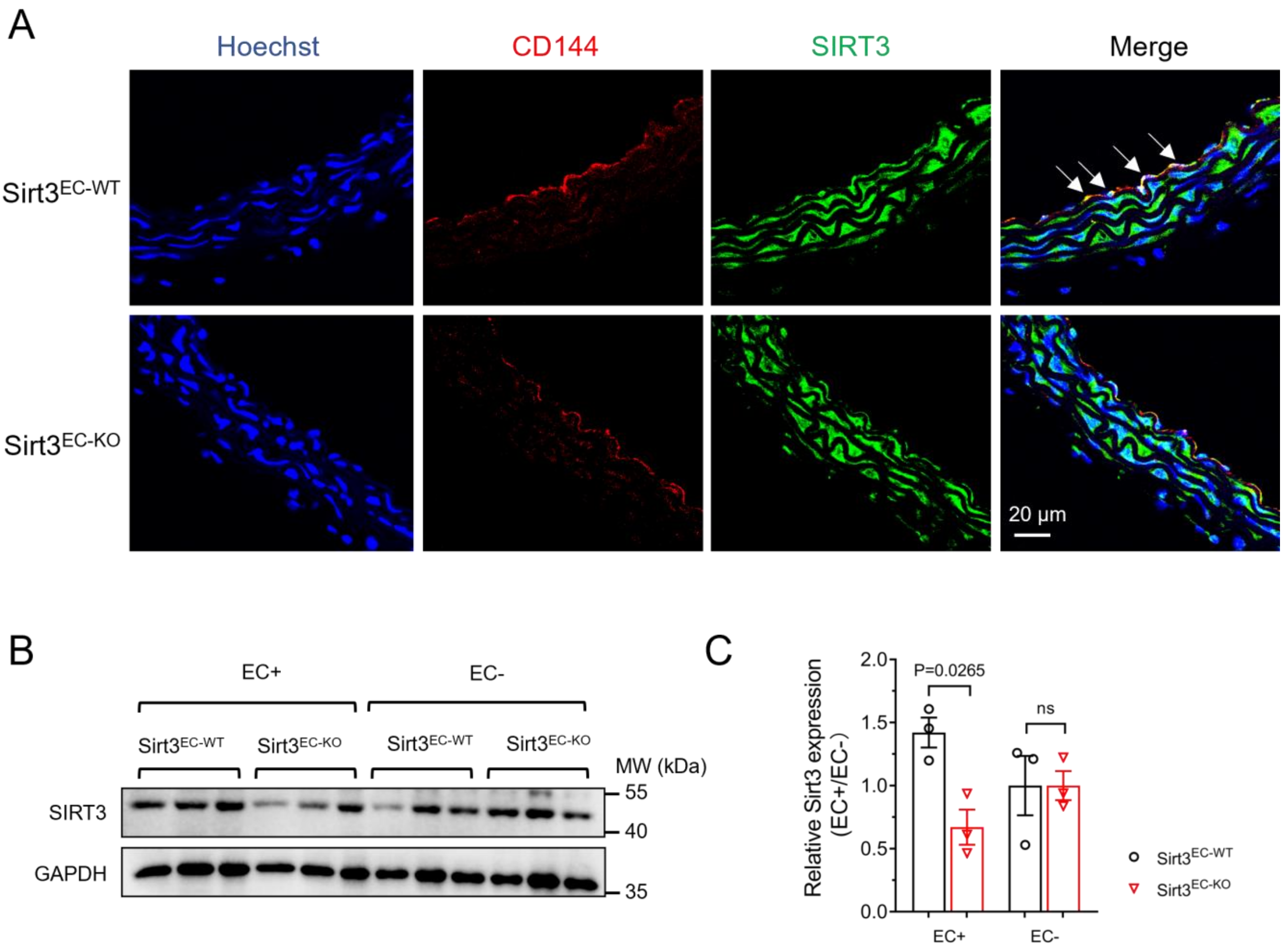

Figure S1. Decreased expression of endothelial Sirt3 in Sirt3$^{EC\text{-}KO}$ mice. A) Immunofluorescence staining of SIRT3 (green) and the endothelial marker CD144 (red) in the aorta cross-sections from Sirt3$^{EC\text{-}WT}$ mice and Sirt3$^{EC\text{-}KO}$ mice. Scar bar = 20 μm. B, C) Validation of endothelial-specific Sirt3 knockout in aortas from Sirt3$^{EC\text{-}WT}$ and Sirt3$^{EC\text{-}KO}$ male mice. Representative Western blot images B) and statistical analysis C) of Sirt3 expression in endothelial-intact (EC+) and endothelial-denuded (EC−) aorta. Data are expressed as mean ± SEM. Statistical significance was calculated by one-way ANOVA followed by Tukey's multiple comparisons test for more than two samples.

D,E

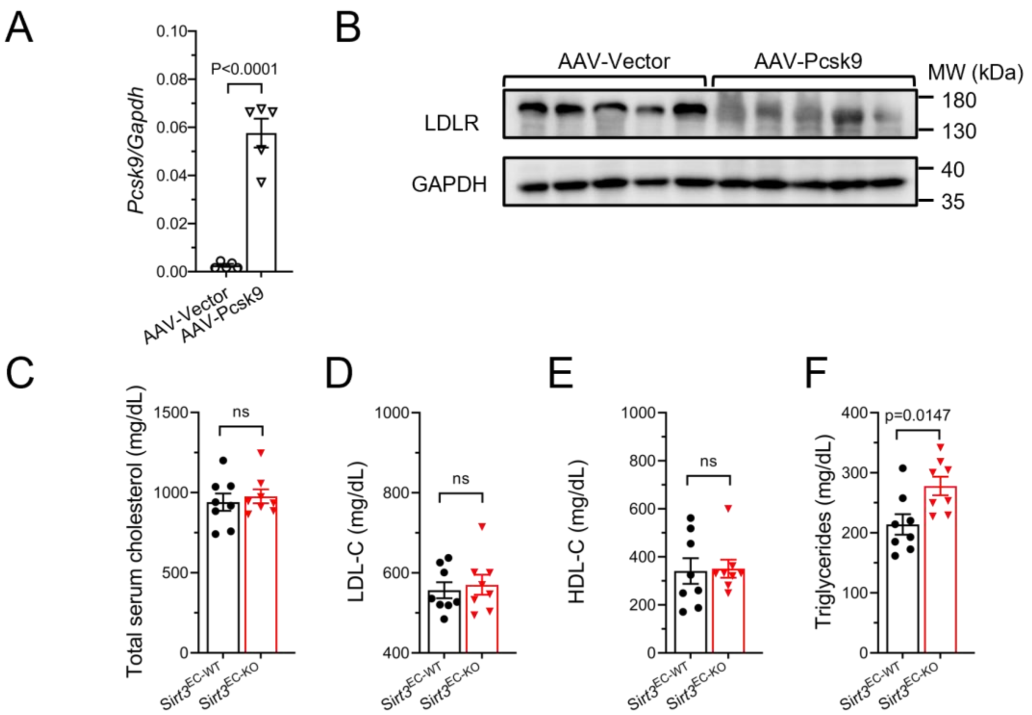

Figure S2. AAV-Pcsk9-mediated knockdown of LDL receptor in mice. A, B) qPCR results for mRNA expression of *Pcsk9* A) and western blot results for protein expression of LDLR B) in livers from C57 mice injected with AAV-Pcsk9 for 12 weeks. *n* = 5 per group. C-F) Total cholesterol C), LDL-C D), HDL-C E) and triglyceride F) levels in the serum from Sirt3$^{EC\text{-}WT}$ and Sirt3$^{EC\text{-}KO}$ mice were fed with HCD for 12 weeks. *n* = 8 per group. Data are expressed as mean ± SEM. Statistical significance was calculated by Student's t-test for comparison between two samples.

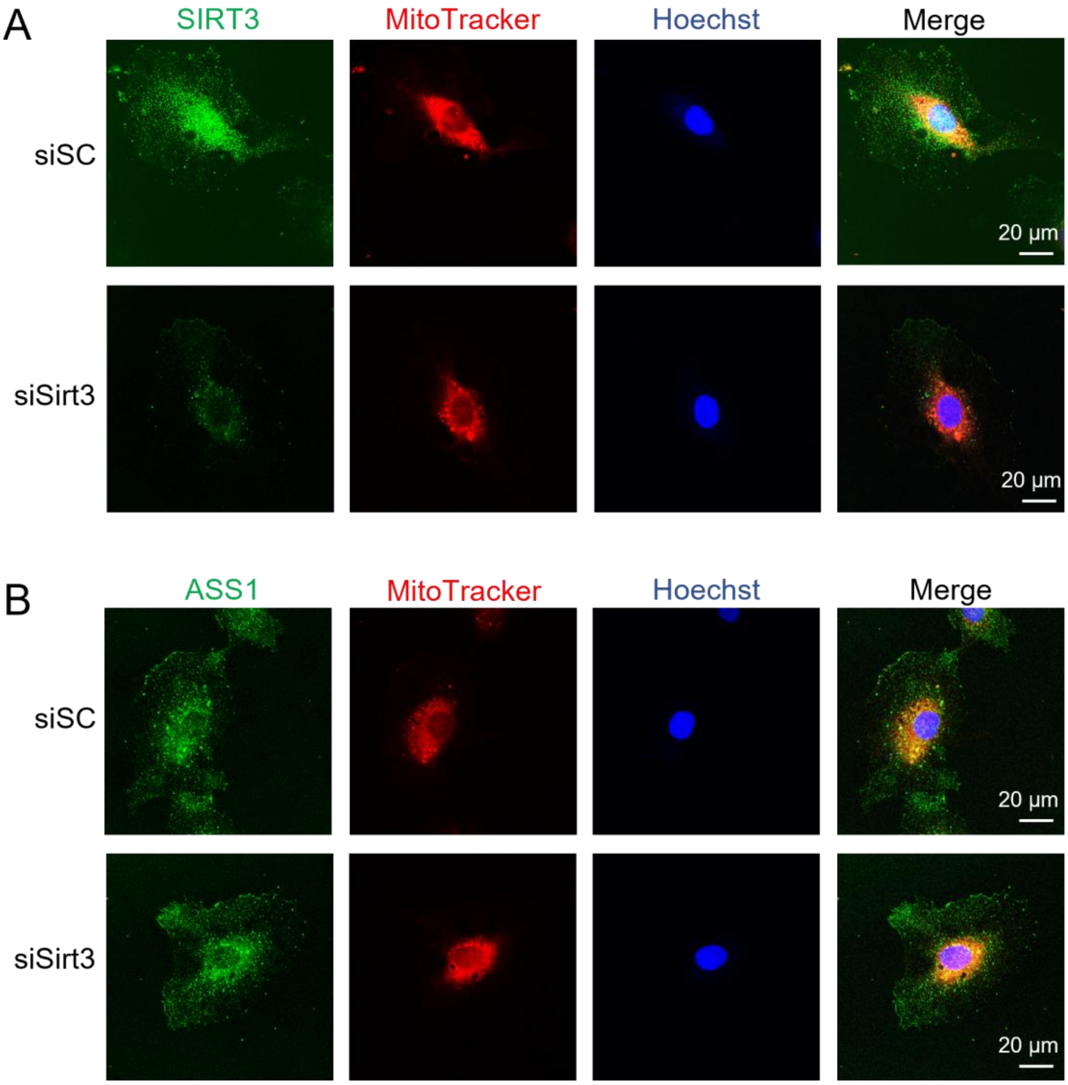

Figure S3. Silencing of SIRT3 in HUVECs did not affect the expression of ASS1 in HUVECs. A, B) Immunofluorescence staining of SIRT3 A) and ASS1 B) in HUVECs transfected with siSC or siSirt3. MitoTracker (red) was stained to indicate mitochondrial localization. Scar bar = 20 μm.

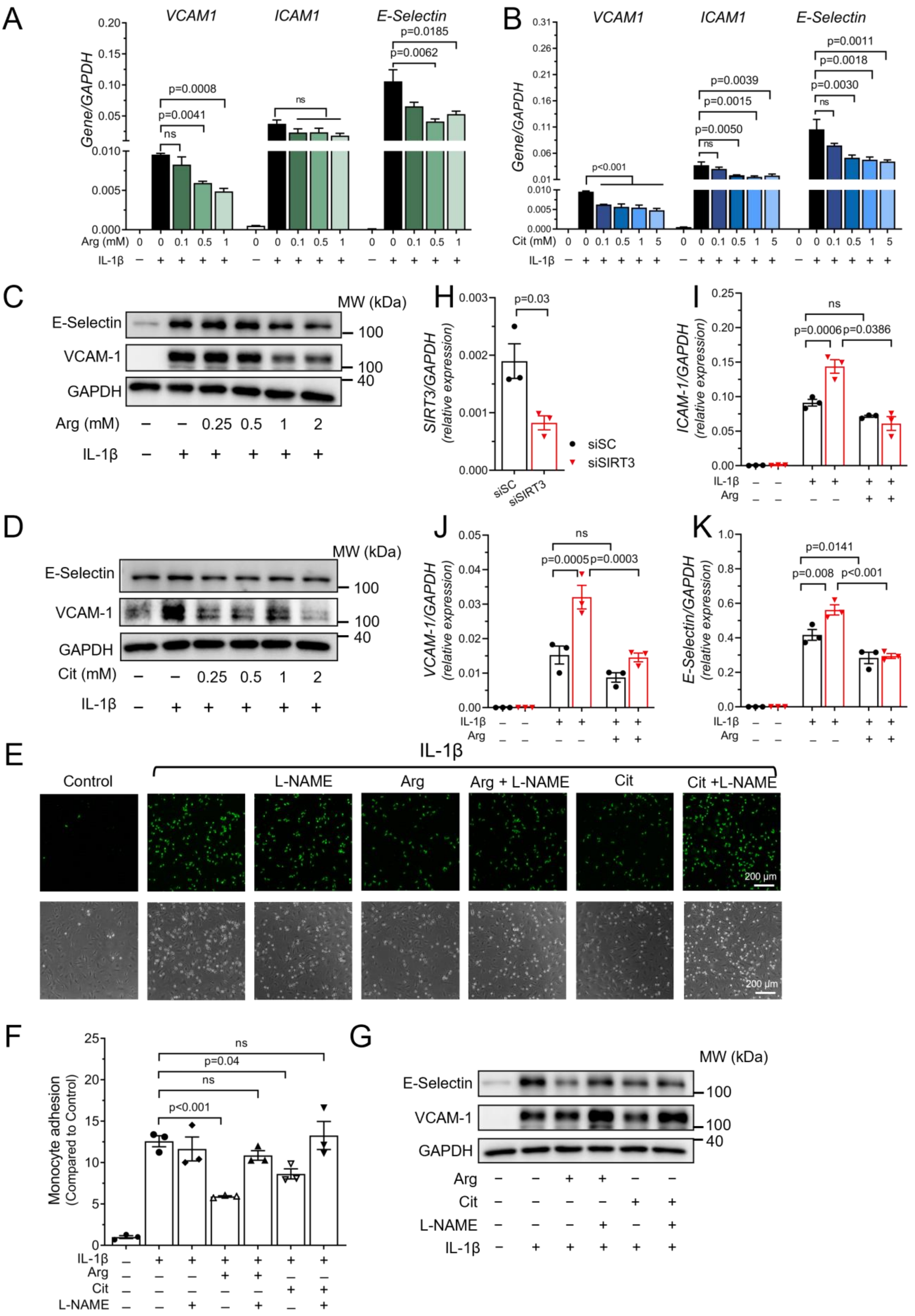

A
VCAM1
ICAM1
E-Selectin
Gene/GAPDH
p=0.0008
p=0.0041
ns
p=0.0185
p=0.0062
Arg (mM)
IL-1β
B
Cit (mM)
p<0.001
p=0.0039
p=0.0015
p=0.0050
p=0.0011
p=0.0018
p=0.0030
C
E-Selectin
VCAM-1
GAPDH
MW (kDa)
Arg (mM)
D
Cit (mM)
H
SIRT3/GAPDH
(relative expression)
p=0.03
siSC
siSIRT3
I
ICAM-1/GAPDH
p=0.0006
p=0.0386
J
VCAM-1/GAPDH
p=0.0005
p=0.0003
K
E-Selectin/GAPDH
p=0.0141
p=0.008
p<0.001
E
IL-1β
Control
L-NAME
Arg
Arg + L-NAME
Cit
Cit +L-NAME
200 µm
F
Monocyte adhesion
(Compared to Control)
p=0.04
p<0.001
Cit
L-NAME
G

Figure S4. L-Arginine and L-Citrulline attenuate endothelial inflammation. A,B) qPCR results of mRNA levels of *VCAM-1, ICAM-1,* and *E-Selectin* in HUVECs treated with Arg A) or Cit B) in a dose-dependent manner as indicated in the figure combined with IL-1β. C,D) Western blot results of VCAM-1, ICAM-1, and E-Selectin in HUVECs treated with Arg C) or Cit D) in a dose-dependent manner combined with IL-1β. E,F) Representative images E) and statistical analysis F) of CSFE-labeled THP-1 cells (Fluorescence microscopic images, green) adhering to HUVECs. HUVECs were treated with L-arginine (Arg, 1 mmol L^{-1}), L-citrulline (Cit, 1 mmol L^{-1}), L-NAME (100 µmol L^{-1}), IL-1β (10 ng mL^{-1}), alone or in combination. Scar bar = 200 µm. G) Western blot results of E-Selectin, VCAM-1 in HUVECs treated with Arg (1 mmol L^{-1}), Cit (1 mmol L^{-1}), L-NAME (100 µmol L^{-1}), IL-1β (10 ng mL^{-1}), alone or in combination. H-K) The mRNA levels of *VCAM-1, ICAM-1,* and *E-Selectin* in HUVECs transfected with siSC or siSIRT3 and treated with Arg (1 mmol L^{-1}) combined with IL-1β (10 ng mL^{-1}). Statistical significance was calculated by by Student's t-test for comparison between two samples and one-way ANOVA followed by Tukey's multiple comparisons test for more than two samples.

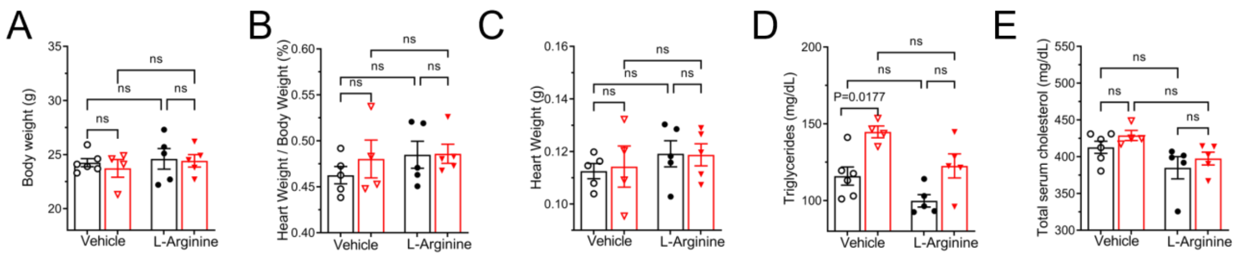

Figure S5. Body weight, heart weight, body weight/ heart weight ratio and lipid profile in the serum from Sirt3$^{EC\text{-}WT}$ and Sirt3$^{EC\text{-}KO}$ mice supplemented with vehicle or L-arginine. A-C) Quantification of body weight A), heart weight B), and body weight/ heart weight ratio C) in Sirt3$^{EC\text{-}WT}$ and Sirt3$^{EC\text{-}KO}$ mice supplemented with vehicle or L-Arginine. D,E) Total cholesterol D) and triglyceride levels E) in the serum from Sirt3$^{EC\text{-}WT}$ and Sirt3$^{EC\text{-}KO}$ mice supplemented with vehicle or L-Arginine. n = 4-6 per group. Data are expressed as mean ± SEM. Statistical significance was calculated by one-way ANOVA followed by Tukey's multiple comparisons test for more than two samples.

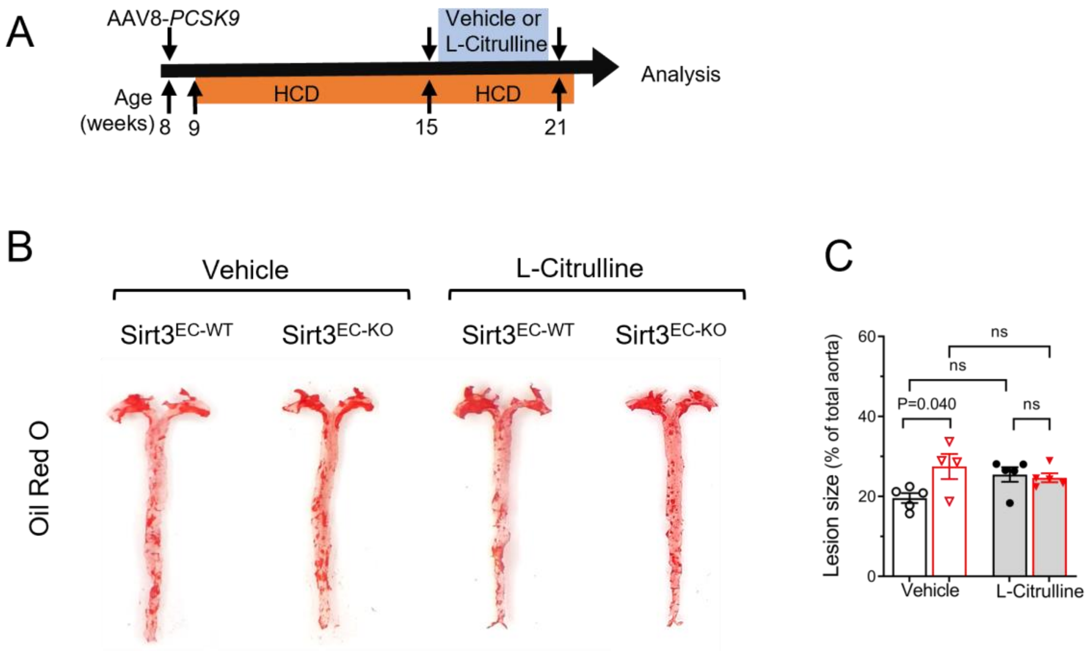

Figure S6. L-Citrulline supplementation has no beneficial effect on atherosclerotic lesion development. A) Schematic experimental outline for the supplementation of L-citrulline in atherosclerotic mouse. $Sirt3^{EC\text{-}WT}$ and $Sirt3^{EC\text{-}KO}$ mice were injected with AAV8-mPCSK9 (2.5×10^{11} vg $mice^{-1}$) via tail vein and fed with HCD for 6 weeks, the mice were subsequently randomly divided into two groups, the vehicle (water) group, and L-citrulline (0.5 % in drinking water) group and fed with HCD for another 6 weeks at the same time. B,C) Representative images B) and statistical analysis C) of en face Oil Red O staining of the aorta. n = 4-6 per group. Data are expressed as mean ± SEM. Statistical significance was calculated by one-way ANOVA followed by Tukey's multiple comparisons test for more than two samples.
